# Peer-to-peer counselling and emotional guidance on infertility in Britain and Belgium (1970s–1980s)

**DOI:** 10.1136/medhum-2022-012505

**Published:** 2023-06-28

**Authors:** Yuliya Hilevych, Tinne Claes

**Affiliations:** 1 History, University of Groningen Faculty of Arts, Groningen, The Netherlands; 2 Cultural History since 1750, KU Leuven Faculty of Arts, Leuven, Belgium

**Keywords:** infertility, History, medical anthropology, reproductive medicine

## Abstract

In the wake of sexual and reproductive health counselling in postwar Western Europe, emotional guidance on infertility was as yet neither readily recognised nor available. In this article, we show that in Britain and Belgium, infertile couples themselves identified the need for systematic emotional guidance on their infertility experiences. They set up self-help support groups to provide counselling on infertility in their respective countries. Originally formed by heterosexual, white, middle-class couples, who were childless due to infertility, these support groups were cautious—rather than affirmative—of reproductive technologies to aid conception. In their view, these technologies were not readily available and did not work for everyone. In this social climate, systematic interactions with peers sought to provide emotional guidance to destigmatise infertility and accept childlessness. This emotional guidance was grounded in the contemporary psychological literature—on grief, mourning and other emotions—that the support groups applied to infertility experiences.

We suggest that these groups could be seen as among the first—in their respective countries and arguably within Europe—to offer infertility counselling through a peer-to-peer format, which is today recognised as a crucial part of professional infertility counselling provision. In this light, our findings uncover previously unseen connections between grassroots support groups, infertility counselling and emotional guidance in the period before infertility counselling was professionalised in Britain and Belgium. Our analysis is based on various archival and published sources as well as oral history accounts, many of which have not been analysed before. Our findings contribute to the history of sexual and reproductive health, history of self-help, history of counselling, and history of emotions.

## Introduction

‘The childless gain some kind of release and confidence from discussing their experiences together’

~ the National Association for the Childless (NAC) (Houghton and Houghton 1977, 6)

‘If you don’t speak, you drift apart’

*~* SARA, self-help group for childless couples (Het Nieuwsblad, 1991, 14–15)

These two quotations vividly illuminate the crucial role of systematically talking through emotions— and doing so with peers—in the experience of infertility, especially infertility leading to involuntary childlessness.1 The quotations come from grassroots support groups that provided counselling on infertility in Britain and Belgium during a period when this type of sexual and reproductive health counselling was neither readily available nor professionalised as yet. In Britain, the National Association for the Childless (NAC) was an initiative that provided counselling on infertility from 1976 and throughout the 1980s. In Belgium, SARA was established as a group for married couples in 1989, providing counselling throughout the 1990s. SARA’s work was preceded by ad hoc short-lived infertility groups supported by the general self-help initiative *Trefpunt Zelfhulp* (Contact Point Self-Help) in 1983.

This type of emotional support developed from the bottom up. The work of NAC and SARA was also novel at the time in their respective countries. Both self-help support groups developed approaches to provide systematic infertility counselling that was grounded in psychological management of grief, mourning and other emotions. In this way, these groups wanted to help couples move through the emotional experience of infertility in a systematic way. Today, this type of systematic emotional guidance is central to infertility counselling (Gameiro and Boivin 2017), which aims ‘to mitigate the physical, emotional and psychosocial consequences of infertility’ (Zegers-Hochschild *et al.* 2017, 1795). Peer-to-peer infertility counselling through support groups is also facilitated by fertility clinics and independent non-profit organisations, such as Fertility Network UK in the UK and De verdwaalde ooievaar (The Lost Stork) in Belgium. In this article, we historicise and illuminate the roots of this systematic emotional approach to infertility counselling in Britain and Belgium, and the role that infertility support groups played in the process. The concept of peer-to-peer counselling, which is used in infertility counselling today to distinguish it from professional counselling, was not used by the initiatives we examine in this article. Nevertheless, following this concept permits us showing how the notion of infertility counselling was interwoven with the practice of systematic peer-to-peer interactions, when the former was still underdeveloped in Europe.

Histories of sexual and reproductive health contain extensive accounts of self-help and emotional counselling on birth control (eg, Cook 2004; Fisher 2006; Rusterholz 2020) and abortion (eg, Sheldon *et al*. 2022; Hoggart 2015; Joffe 2013) in postwar Europe. More recently, historical studies on infertility and reproductive technologies have also flourished (Davis and Loughran 2017; Hopwood, Flemming, and Kassell 2018; Marsh and Ronner 2019; Szreter 2019). Within this literature, scholars have attempted to shed light on professionals’ attitudes towards the psychological aspects of infertility in the recent past (Gameiro and Boivin 2017; Lewis 2010; Balen 2002; Claes 2022b). Anthropological and sociological studies have also examined contemporary support groups and their emotional guidance, for example in pregnancy loss (Layne 2003), egg donation and freezing (Inhorn 2021; Konrad 2005), coping with infertility treatment (Gerrits 2016; Thompson 2005) and its failures (Throsby 2004). Altogether, these studies shed light on the multifaceted character of various aspects of professional guidance and emotional experiences of infertility. However, this growing body of historical and social sciences scholarship has so far paid little attention to the historical roots of infertility counselling. Indeed, how people experiencing infertility were counselled and what roles emotions played in infertility counselling in the past have remained unexplored.

In this article, we examine initiatives that—we will argue—were among the first to provide infertility counselling in Britain and Belgium and in Europe in general. NAC and SARA were originally formed by heterosexual, white, middle-class couples, who were childless due to infertility. NAC and SARA did not know each other. Nevertheless, they both developed in a social climate when infertility was becoming more visible and the importance of providing infertility treatment was becoming more recognised (Pfeffer 1993; Beers 2022; Claes 2022a). Britain and Belgium in the postwar period were characterised by distinct religious and political cultures. However, in neither country was access to infertility treatments yet readily available to involuntary childless couples. As we have shown elsewhere, even religious actors in the 1970s and 1980s were becoming open to the need for infertility treatments and counselling in both countries (Claes and Hilevych 2022).

Not only do we shed light on the origins of support groups on infertility, but also on the peculiarities of the emotional guidance provided by these groups. We look at support groups as ‘emotional communities’—in that they were social groups specifically convened to share the expression of particular feelings (Rosenwein 2006, 2; Plamper 2015, 69). In our analysis, we conceptualise the ways these support groups shared, and indeed shaped, the emotional norms and rules around infertility leading to involuntary childlessness. We refer to these emotional norms as emotional guidance, because these groups aimed at helping to guide couples in their journeys of coping with infertility. This emotional guidance of infertility was situated in a wider social climate of a ‘child-centred’ society, as perceived by both groups in Britain and Belgium, as we will show below.

Against this background, as we will demonstrate, these initiatives’ counselling approaches on emotional guidance around infertility were strikingly similar. Both NAC and SARA sprung from the central idea that infertility treatments were rarely successful, and that counselling had to account for this. Today, these narratives on ‘unsuccessful’ assisted reproductive technologies are often seen as marginal (Throsby 2004); scholars have repeatedly shown how in vitro fertilisation (IVF) (Franklin 1997) and egg freezing (Inhorn et al. 2022) have become ‘hope technologies’ for those who experience infertility. Our study will add a new historical perspective on the practical-medical notions of ‘acceptance’ and ‘counselling’ as well as on emotional encounters with ‘hope’, ‘grief’ and other emotions around infertility. Our study contributes to the scholarship on sexual and reproductive health history, as well as to the history of self-help, history of counselling and history of emotions.

The article draws on published materials both about and by these initiatives and on various archival sources documenting the work of the organisations, many of which have not been analysed before. The published materials include self-help manuals and brochures, as well as newspaper articles and radio coverage surveying the work of these groups. In addition, we have also drawn on four oral histories—two with the founders of SARA in Belgium and two with people who worked with NAC in Britain.2

This article consists of three parts. In the next part, we discuss how infertility was historically positioned in sexual and reproductive health counselling in postwar Britain and Belgium. Following this, we compare how grassroots initiatives started offering infertility counselling through the peer-to-peer format. Lastly, we turn to the analysis of the emotional guidance offered by these groups: how were the infertile advised to manage their emotions? What were seen as helpful ways to ‘cope’ with infertility and involuntary childlessness?

## Infertility within sexual and reproductive health counselling in postwar Britain and Belgium

In Britain, counselling on issues concerning sexuality and reproduction had been carried out since the interwar years by non-governmental and charitable initiatives, such as the National Marriage Guidance Council (NMGC) and the Family Planning Association (FPA) (Hall 2000). The NMGC (Lewis, Clark, and Morgan 1992) and later also Catholic Marriage Guidance Counselling (Harris 2015) provided counselling for marital relationships. Their work was guided by the ideal of the companionate marriage3 and focused on the issues of marital stability, prevention of divorce, mental health within the family and sexual difficulties (Chettiar 2016; Irwin 2009; Cook 2005). However, there is no historical evidence suggesting that sterility, infertility and childlessness were addressed as separate matters by NMGC.

The FPA, on the other hand, was explicit about the fact that sterility and infertility were part of their work. Scholars suggest that since the interwar period at least, the FPA had adopted a holistic approach to reproductive health (Leathard 1980; Pfeffer 1993; Rusterholz 2020; Beers 2021). In practice, this implied that the FPA had been providing medical guidance on birth control, as well as on sterility and infertility, since the interwar years. Some specific FPA doctors also provided emotional counselling on sexual difficulties, which for some couples could lead to being unable to conceive and have a child. As Rusterholz (2019) has shown, among such doctors was Joan Malleson, who provided sexual counselling in the 1930s, 1940s and 1950s at the FPA clinic in London. Malleson’s work addressed sexual difficulties and could be seen as the root of emotional counselling on sterility in Britain (Rusterholz 2020). After Malleson’s death, the psychiatrist Michael Balint, from the Tavistock Clinic, took over in 1957. Balint’s work with FPA was directed more towards training general practitioners in sexual counselling (Irwin 2009), which formalised psychosexual training within the FPA (Rusterholz 2020). However, as far as historians are aware, the FPA did not introduce any systemic emotional guidance provision directly targeting for couples experiencing sterility and infertility.

The provision of sexual and reproductive health counselling was similar in postwar Belgium, with the exception that religion and specifically Catholicism played a crucial role in these services. Several family planning centres were established in the late 1950s, fuelled by public debates on birth control and the purpose of (Roman Catholic) marriages.4 As Belgium was a ‘pillarised’ society at the time (Post 1989), there were several family planning centres that provided counselling services according to their own values and criteria, influenced by the religious or philosophical convictions of their given pillar (Catholic, Liberal or Socialist). Even though the Catholic pillar was the largest, more ‘controversial’ topics—including birth control and abortion, as well as homosexuality and assisted reproduction—were more frequently and positively addressed within secular organisations (Trommelmans 2006).

This is not to say that Catholic organisations did not touch on these issues. On the contrary, recent studies have indicated that contraception and abortion were discussed within Catholic family planning centres and that there was an increasing tendency to disregard the Vatican’s opinion on these matters after the controversial encyclical *Humanae Vitae* appeared in 1968 (Crosetti 2020; Dupont 2018; Masquelier 2021). From a case study on Catholic doctors’ views on assisted reproduction, it appears that similar dynamics were at play when it came to artificial insemination and IVF (Claes 2021). Although the Vatican rejected ‘artificial’ conception—much like ‘artificial’ contraception—many Catholic doctors did offer these treatments and presented them as moral. However, due to the lack of research on the topic, we still do not know whether and, if so, how counsellors in family planning centres handled these matters.

We do know that, independently of their ideological colour, all family planning centres in Belgium were mainly aimed at married couples. They propagated the importance of a harmonious sexual life, which, like in Britain, was underpinned by the ideal of a ‘companionate marriage’ (Gevers 2014). Studies that have examined the work of these centres indicate that their focus was on the topics of sex education, contraception and abortion, with therapy and counselling around these topics only indirectly touched on (Carlier, Deven, and Triest 1990; Crosetti 2017). Although the provision of medical advice and counselling on infertility was not their main priority, the conserved archival materials of family planning centres do contain various newspaper cuttings, publications and letters about infertility and reproductive medicine, which suggests that couples looking for help did go there for advice on enhancing their fertility.5 In the 1980s, family planning centres also published brochures about possible ‘alternatives’ to involuntary childlessness, such as adoption, foster care, various infertility treatments, or even divorce .6 From the source materials, however, it is unclear whether family planning centres were offering specific emotional guidance and counselling for infertility or for any sexual difficulties associated with it.

Neither was infertility the primary focus of feminist movements at the time in Britain and Belgium. Historians suggest that the British women’s liberation movement (WLM) in the 1960s, 1970s and 1980s marginalised and often ignored infertility. Specifically, there are historical accounts suggesting that WLM typically constructed motherhood as a choice, which is why the desire for a child—and certainly the biological inability to conceive—was not a priority for them (Loughran 2017). Even in later discussions, various British WLMs were also largely against new reproductive technologies such as IVF (Beers 2022).

At the same time, scholars do suggest that in the 1970s, at the grassroots level, some feminist health organisations that had originally focused on birth control started to provide referrals for infertility (Olszynko-Gryn 2019). But these were marginal rather than common cases, and it is not clear whether they provided any emotional support. Also, the British edition of *Our Bodies Ourselves* (Phillips and Rakusen 1978, 498) included a section on ‘Exceptions to pregnancy and childbirth’ that covered infertility and miscarriage among other topics. The first explicitly feminist handbook *The experience of infertility* (Pfeffer and Woollett 1983) went even further by illustrating actual lived experiences of infertility, mainly citing women’s testimonies. However, and perhaps not surprisingly, both handbooks cited the NAC—one of the organisations analysed in detail in this article—as the main contact point for further advice and emotional guidance on infertility.

Similarly, within the Belgian context, the women’s movement paid comparatively little attention to the topic of infertility. A strong focus on the provision of contraception and abortion (the latter was illegal until 1990) appears to have overshadowed other women’s health topics (Gijbels and Wils 2021). Indeed, scarce attention was paid to infertility in publications by the women’s movement. For instance, *Les Cahiers du GRIF*, one of the main French-speaking feminist periodicals, published only one article about assisted reproduction in the 1970s (Gallez 1974). To give one more telling example, when the president of *Vrouwen Overleg Komitee* (the Women’s Negotiation Committee), an important feminist organisation in Flanders, was interviewed about the topic of infertility treatments in 1994, she replied that ‘there are individuals within the feminist movement who don't think it’s a good idea, but in general it hasn't been discussed yet’.7

In the sphere of medical provision of infertility treatments and examinations, emotional counselling also occupied an ambiguous role in both contexts. In Britain, provision of infertility treatment was not universal. Until the 1980s at least, it depended on individual doctors, who provided these treatments first through private or charitable clinics such as the FPA, and later through the National Health Service (Pfeffer 1993; Rusterholz 2020; Beers 2021). Individual doctors could possibly have decided to screen couples for suitability for the treatments.8 While these checks on patients’ suitability for treatment were not universalised, in the literature from the 1970s and 1980s, they were commonly referred to as counselling (Snowden and Mitchell 1981, 54–55). The ambiguity is that this type of screening-counselling did not imply emotional guidance of the patients, especially during or after the treatments. Indeed, contemporary literature on infertility counselling in Britain suggests that until the Warnock report (1984), emotional counselling of patients undergoing infertility treatment was not seen as part of professional infertility care (Jennings 1995).9 Although limited, however, there is scarce evidence that some social workers in fostering and adoption provided emotional counselling on the social experience of infertility to the childless in the 1970s and 1980s (Monach 2003). But this was not a universal provision and again depended on individual social workers.

In Belgium, medical provision of infertility treatment started in the postwar years (Nys 2017; Claes 2021). However, mental health professionals from various professional backgrounds—psychiatrists, psychologists, sexologists, therapists and social workers—only became involved with fertility clinics in the 1970s. Moreover, their focus initially was not on helping patients to deal with the distress caused by infertility, but on selecting suitable patients who in their view would be able to cope with treatments and become adequate parents. Thus, their primary role was gatekeeping: they decided who could undergo treatment and under which conditions (Claes 2022b). As their focus was on selecting and screening procedures, mental health professionals mainly met intended parents before—and not during or after—treatment. Therefore, coping with permanent childlessness due to infertility remained a blind spot in their work, which was more about managing expectations and feelings during treatment when couples were still hoping to become pregnant. Even for couples who were still trying to conceive through medical treatment, fertility clinics did not play a big part in the emergence of peer-to-peer counselling. On the contrary, when Dutch fertility doctors, during a meeting on the psychological consequences of donor insemination in 1979, tried to convince their Belgian colleagues of the importance of ‘sharing emotions’ for the ‘mourning process’ of infertile couples, the Belgians answered that ‘no one would come to such a group’.10

Infertility counselling, as a means of emotional guidance for couples undergoing treatment, was therefore not yet recognised within the medical sphere in postwar Britain and Belgium. Within marriage and family planning organisations, as well as feminist movements, infertility was treated as part of other sexual and reproductive concerns, and not as a subject in its own right. In this light, the initiatives that started to provide infertility counselling in the late 1970s in Britain and the mid-1980s in Belgium could be seen as a response to the lack of this type of counselling. These initiatives also draw on the aforementioned advancements in professional counselling and self-help. Additionally, it was the context of the more frequent provision of medical infertility treatments where the need for infertility counselling was becoming apparent, as we show in the next section.

## Self-help groups and the establishment of peer-to-peer counselling on infertility

### The NAC (Britain)

In Britain, the first self-help support group that focused specifically on infertility and made itself public was set up in Birmingham in 1974; it was founded by Peter and Diane Houghton (Houghton and Houghton 1977). Initially, this support group functioned on an informal basis in the Birmingham Settlement—a secular, charitable organisation providing various types of community welfare in the city. In 1976, the Settlement held the first national conference for the childless, where it was decided to form a nationwide initiative: the NAC. The idea was that while the NAC headquarters were in the Birmingham Settlement for practical reasons, a network of infertility self-help groups across the country would be set up as grassroots initiatives (Birmingham Settlement 1976).

In 1979, local initiatives under the umbrella of the NAC were set up in all major English towns, as well as in Cardiff in Wales and Glasgow and Edinburgh in Scotland (Birmingham Settlement 1979). The NAC also positioned itself as a national pressure group to lobby for better access to infertility treatments, adoption and fostering services, as well as better inclusion of childless people in a ‘child-oriented’ society (Houghton and Houghton 1977). While in 1979, a new pressure group, CHILD, was set up with a view to focusing specifically on access to infertility treatments (The Observer 1979, 40), the NAC’s role has been seen as pivotal in setting up local infertility counselling in England and eventually across Britain (Monach 2003).11

The need for a multifaceted association that would focus not only on raising awareness but also on providing systematic counselling around infertility originated with Peter and Diane Houghton. The Houghtons were a heterosexual, white, university-educated, middle-class couple, who themselves had been born to working-class families; they did not have children due to infertility (Houghton and Houghton 1977; Houghton and Houghton 1984). Peter Houghton was a trained psychologist and worked as a counsellor and adviser at Birmingham Settlement, which was the NAC’s headquarters at the time. Diane was pursuing her university master’s degree and teaching English. Before the NAC was set up in 1976, Peter was already the Director of the Settlement (Birmingham Settlement 1976). It was in this role that he encountered and worked for several years with childless couples, both individually and in small groups at the Settlement.

In their account of the NAC’s emergence, the Houghtons stated that Peter encountered couples who were ‘in reasonable health, financially secure, with a stable relationship with another adult, and socially and career-wise they may have appeared competent and fulfilled’, but were ‘suffering from the sense of pointlessness or meaninglessness’ (Houghton and Houghton 1977, 6). It soon became clear to the Houghtons that ‘the childless gain some kind of release and confidence from discussing their experiences together’ (Houghton and Houghton 1977, 6). They also saw ‘the therapeutic possibilities of such an Association for those unable to have children’ (kNACk 1977, 1). However, the Houghtons’ personal experiences of infertility were a crucial catalyst for setting up the NAC. These experiences were discussed in their two handbooks (Houghton and Houghton 1977; Houghton and Houghton 1984) aimed at childless people, which included the testimony ‘One woman’s story’, written primarily by Diane.

After several years of trying to have a child, the Houghtons had decided to adopt. They did not plan to have a medical examination as they ‘did not feel strongly about having a child of their own’ (Houghton and Houghton 1977, 29). However, to be considered for adoption, the Houghtons needed a medical certificate proving the sterility of one or both partners. According to the Houghtons, the doctors did not ask Peter to play much of a role in the medical investigation; it focused on Diane. After all the screenings, however, she was not able to secure a referral for treatment because their specialist ‘did not issue letters that anyone was incapable of having children, unless she were without a womb’ (Houghton and Houghton 1977, 29). As illustrated by this quotation, their personal experiences of infertility were turbulent and quite likely very emotional for the Houghtons. These experiences started off a circle of medical check-ups that the Houghtons referred to in one of their handbooks as the ‘infertility treadmill’ (Houghton and Houghton 1984, 48) —a very emotional process, as will be discussed in the next section. However, after all these examinations, the Houghtons were unable to secure a medical letter proving their sterility (Houghton and Houghton 1977, 29). This meant, as they were told at the time, that they were unlikely to be successful in applying for adoption. Fostering also proved difficult for them at the time when they were writing the two handbooks. However, in the course of their work at NAC and thereafter, they would become foster parents,12 as some accounts suggest, to 11 children in total (Richmond 2007).

The Houghtons began a project to make support around infertility available to more people across the country. More than that, they envisioned creating a network of trained counsellors who would provide emotional guidance in peer settings around infertility, especially when treatments and adoption were not possible (Houghton and Houghton 1977). As we will show below, this counselling work was informed by popular psychology literature at the time. This permitted the Houghtons to establish a systematic approach to counselling, which ad hoc self-help groups would not be able to provide. Because Peter Houghton was also trained in psychology and was the Director of the Birmingham Settlement, he had both the experience and capacity to set up this type of counselling provision at the community level. These factors, as well as his early and persistent communication with the Department of Health and Social Security (Hilevych 2019), would subsequently allow him to secure some additional funding for the provision of infertility counselling, initially through the national network of self-help groups (Birmingham Settlement 1978), and in the early 1980s through the so-called NAC Contacts (Houghton and Houghton 1984). NAC Contacts were people who had not only experienced infertility themselves but were also specifically trained in counselling skills. NAC Contacts were available to counselees by phone and worked on a voluntary basis; some continued to remain in contact even after they had children, as the Houghtons suggested in their handbook.

In the Houghtons’ view, this need for counselling provision was linked to the increasing number of people who were childless due to infertility or other involuntary reasons. For example, the Houghtons suggested that in Britain the proportion of childless people constituted nearly 8% of marriages, or around 2 million people across the UK. Including single people who were childless in midlife—they suggested—the estimate would be even higher, around 10% of the UK population (Houghton and Houghton 1977, 9). Subsequently, the Houghtons defined the childless as those who were unable to have a biological child of their own at some point in life, and who actively sought to have a child or had an emotional impact from failing to have a child (Houghton and Houghton 1984, 12–13). Because of the emphasis on the emotional component, the Houghtons suggested that the medical term infertility was ‘inadequate for these purposes’ (Houghton and Houghton 1984, 13). The Houghtons and NAC used the term ‘childless’ to refer to those who could not have children, and to make a distinction from those who were ‘childfree’.13 They thus referred to childlessness to imply ‘involuntary childlessness’ in the present-day terms. All in all, the Houghtons suggested that it was not just the biological but also the emotional aspect of infertility that led NAC members to come together and seek help from each other. The childless could not belong fully to what the Houghtons called the ‘child-centred’ society, where everyone was expected to want and have children (Houghton and Houghton 1977, 6–7). As such, the focus on belonging and fighting social stigma against childlessness was one of the core aspects of the NAC’s work; they saw infertility counselling as the way to help childless couples, as we will show after discussing the Belgian case.

### Trefpunt Zelfhulp and SARA (Belgium)

In Belgium, self-help support groups that focused on infertility had different roots to those in Britain, yet their focus on infertility counselling placed similar issues on their agendas. The first self-help groups in Belgium were established in the 1980s and had a somewhat hesitant start. When the Flemish umbrella organisation for general self-help, *Trefpunt Zelfhulp* (Contact Point Self-Help), planned their first meeting on ‘the issue of infertility’ in 1984, there were, according to its report, ‘too few people present to really speak of a group’ (Tweemaandelijks Tijdschrift Trefpunt Zelfhulp 1984). Trefpunt Zelfhulp had been established in the wake of international calls for self-help in health. In 1980, the Council of Europe encouraged member states to set up programmes to stimulate patient participation during medical treatment, including self-help groups and patient organisations. The World Health Organisation underlined the importance of self-help in the same year (Branckaerts, Nuyens, and van Wanseele 1982). As a result of these calls, as well as a research report that indicated the need for coordination of existing self-help initiatives, the Flemish Ministry for Welfare, Public Health and the Family decided to fund the organisation in 1984 (Paepe and Nuyens 1984). Self-help hence did not solely emerge from the bottom up but was also stimulated from the top down. The initiatives set up by Trefpunt Zelfhulp were part of a wider movement that sought to enhance patients’ agency and autonomy. This does not mean, however, that the groups supported by this organisation were all patient groups or centred around medical treatment. On the contrary, groups dealt with a wide range of topics, including various diseases and disabilities; there were also peer support groups for single parents, divorcees, parents of deceased children and so on.14

Trefpunt Zelfhulp decided to start with a self-help group for the involuntarily childless in response to numerous requests. In 1983, involuntary childlessness was the most common ‘social problem’ (before divorce and widow/erhood) for which people asked the organisation for help: 4% of all phone calls to the organisation came from involuntarily childless individuals looking for information or support (Paepe and Nuyens 1984). Trefpunt Zelfhulp first encouraged childless couples to start or join self-help groups by placing advertisements in various magazines and through the popular radio show ‘Service Telephone’ (Daegsels 1985). These calls were met with moderate enthusiasm: in 1984, three local self-help groups were established across Flanders, each consisting of approximately 20 couples. However, as these groups mainly relied on one couple’s initiative, they struggled to survive: by the end of 1985, all three groups had ceased to exist (Vandermeulen and Branckaerts 1988). Therefore, they have left scant traces in the historical record.

Throughout the second half of the 1980s, there were similar short-lived grassroots initiatives across Flanders, Wallonia and Brussels. Newspapers of the period contain advertisements for informal meetings of involuntarily childless couples, with the aim of sharing their experiences and helping each other (Gazet van Antwerpen 1984; Gazet van Antwerpen 1989). In the collection of the Belgian Women’s Archive, we also found a bilingual (Dutch and French) poster of a Brussels group called *Alice et les 3 E*, which promised ‘infertile couples’ support and guidance ‘from people who have been through it themselves’.15 It appears that French-speaking Belgians also became members of the French association *Pauline et Adrien*, a group that still exists today, which was established by Chantal Ramogida after having undergone infertility treatment herself.16 From the 1990s onwards, *Centre des nouvelles parentalités* (the Centre of New Parenthoods) (CNP), which was created in 1988 on the initiative of psychologist Caroline Bourg, also began to organise *cycles de rencontres* (‘meeting circles’) for infertile couples, who could discuss their issues under the guidance of a mental health professional. These meetings took place in family planning centres and in fertility clinics.17

However, even when systematic emotional support slowly began to gain importance in professional settings, grassroots groups continued to be established and disbanded. In French-speaking Belgium, there was the group *Bébé notre espoir* (Baby Our Hope), which, to our knowledge, has not left any archival materials. In Flanders, the first self-help group that lasted for more than a year appears to have been SARA, named for the story of Sarah (Sarai) in Genesis, one of the first mentions of infertility in Judeo-Christian traditions. The work of SARA was also different from other support groups, because just as in the case of NAC, it relied on popular psychology literature to formulate a unique counselling approach to infertility, as we will show below.

SARA was initiated by a married couple, Paul Dewickere and Frieda Franck, who were themselves childless and had both trained as pastoral workers. They identified as Roman Catholic. From 1989 onwards, their initiative also became supported by the organisation for pastoral family guidance in Antwerp (Pastoraaltje 1989). Even though SARA as a group in principle was open to everyone, whatever their religious convictions, the counselling offered was clearly inspired by Catholic discourses and ideas. For example, the name of the group, SARA, referred to ‘the biblical ancestress, for whom childlessness was a heavy burden, but who dared to give life with courage and confidence, and therefore was life-giving nevertheless’ (Kontaktblad 1991).

Much like the case of the Houghtons and the NAC, the personal experiences of Paul Dewickere and Frieda Franck very much shaped the work of SARA.18 The founders repeatedly stressed that they wanted to give to others what they had missed themselves, namely emotional guidance and peer support when seeking infertility treatments and coming to terms with childlessness. As Paul Dewickere explained in an interview, they founded SARA ‘because we experienced how liberating it may be to be able to talk about your sadness and problems, and how hard it is to find people who are in the same situation’ (Waes 1991, 27).

SARA linked this need to connect with ‘people in the same situation’ to the general focus on children in society. Even though they did not refer to a ‘child-centred society’, as the Houghtons did, their publications also repeatedly claimed that society was geared towards families with children (Kerk en Leven 1989). Precisely because having children was the norm, so they argued, it was hard to connect to others when you were childless due to infertility. In talks and interviews, Dewickere and Franck therefore emphasised that they wanted to increase the public visibility of infertility, a topic that, in their view, was rarely talked about because having children was ‘institutionalised’ (R.B 1991). In their words, ‘there rests a massive taboo on being childless. After all, you are not like the others’ (Kontaktblad 1991).

Similar to the British example, SARA aimed to break this taboo and to raise public awareness about infertility. In order to make clear that infertility was not an exceptional experience—and far more common than was generally thought at the time—they repeatedly used the statistic that 10% of all marriages remained childless (Pastoraaltje 1989). They also clearly differentiated the involuntarily childless from the voluntarily childless, a distinction that was becoming much more pronounced in the late 1980s and 1990s. The voluntarily childless group, in SARA’s view, was a lot less numerous: ‘only 1 to 2 per cent […] of couples who are childless are so by choice’ (R.B 1991). At the same time, they also emphasised that those unmarried could be considered to be involuntarily childless:

‘Remaining unmarried can also be “forced” on you by all kinds of circumstances: long-term care for parents or relatives, a physical or mental disability, a different sexual orientation or simply for no apparent reason. […] But with whom do they get the opportunity - if they wish - to discuss their childlessness?’ (Dewickere and Franck 1994, 98–99).

In practice, however, SARA mainly consisted of married couples. As they explained in a recent oral history interview, Dewickere and Franck were mostly contacted by involuntarily childless women, but always urged them to bring their partners to counselling. In their own words: ‘We always told them if they called, it is better if you *both* come [the wife and the husband], but you are also allowed to come by yourself.’19 This emphasis on the well-being of the married couple, rather than the individual, was typical of Catholic counselling of the time.20

Rendering infertility visible was key in the advice SARA gave to people with children. In the chapter ‘People with and people without children’ in their autobiographical book, they [Bibr R22]gave advice on how to behave towards a childless man or woman. For example, they urged people to create room for open conversations about childlessness, not to monopolise the conversation with stories about their own children, and not to wish people ‘a baby’ on New Year’s Eve, because this could be painful for the infertile (Dewickere and Franck 1994, 81–95). In the next chapter, they made a plea for making the childless visible in every part of society, including, for example, the Roman Catholic Church:

‘Also in sermons and intercessions we hardly ever hear anything about *our* situation. Therefore we get the impression that in the Church only the classical family with children is addressed and oh-so-rarely the many others: the childless families, the single-parent families, the widowed, the divorced, the singles…’ (Dewickere and Franck 1994, 99).

Even though SARA was clearly influenced by the Catholic pillar, the advice they gave did not differ from NAC as much as one might expect. Even a secular organisation like NAC was critical of infertility treatments at the time. In short, there were different origins in the way grassroots infertility counselling was organised in Britain and Belgium. However, as we show in the next section, emotional peer-to-peer guidance was made central to NAC’s and SARA’s counselling content. As there are no accounts of the specific emotional approaches to infertility counselling provided by the groups supported by Trefpunt Zelfhulp in Belgium, probably because they were ad hoc and short-lived, in the next section we analyse approaches to infertility counselling by the NAC in Britain and SARA in Belgium.

## Emotional guidance and coming to terms with infertility: comparing NAC and SARA

For NAC and SARA, the difficulties of belonging in a child-centred society were a crucial similar aspect that guided their work. However, NAC’s and SARA’s approaches went a step ahead of the ad hoc self-help. Both initiatives developed their own psychological methods for helping people come to terms with infertility, especially when it resulted in childlessness. The founders of these initiatives—the Houghtons (NAC) and Dewickere and Franck (SARA)—adopted approaches from other fields of psychology to address infertility in a systematic way. They also used the terms as counselling and therapy to describe their work. As we show below, for them the central aspects of infertility counselling were first recognising and processing emotions of grief and mourning, and eventually destigmatising infertility and accepting childlessness.

### Recognising emotions of grief and mourning around infertility

In Britain, the Houghtons’ work with the Settlement’s infertility self-help groups (1974) and from 1976 onwards with NAC became the focus of their first handbook, ‘*Unfocused grief*’, published by the Birmingham Settlement in 1977. In the handbook, they set out clearly that the goal of the Association was to fight against stereotypes associated with childlessness. They felt that childless people were seen as sad, lonely, pathetic, bitter, dull, lacking vitality and meaning, and overendowed with material benefits (Houghton and Houghton 1977). The very idea of ‘unfocused grief’ in the handbook title aimed to capture the specific emotional concerns of the childless—the experience of infertility.

They developed the idea of ‘unfocused grief’ based on the book ‘*Bereavement – studies of grief in adult life*’ by Dr Colin Murray Parkers, published in 1975. Through a detailed comparison with the bereavement linked to death discussed by Parkers, the Houghtons suggested that the emotional state of childlessness due to infertility could also be seen as ‘grief’. As in the process of bereavement, the feeling of loss was also present and relevant to a childless person’s experience. However, unlike when losing someone, grief was unfocused for the childless. The loss was hard to grasp because the grief was for the experience of children and parenthood that the childless never had (Houghton and Houghton 1977).

The Houghtons’ conceptualisation of grief was similar to the theorisations presented by Barbara Eck Menning, a founder of the US organisation Resolve (1974).21 Like the Houghtons, Menning had published her first self-help handbook, ‘*Infertility: A Guide for the Childless Couple*’, also in 1977. Menning suggested that infertility was an emotional state that was linked to a range of feelings, including anger, shock, isolation, denial and grief (Menning 1977). Similar to the Houghtons, she adopted a framework of bereavement. However, where the Houghtons referenced Parkers, Menning based her work on that of Elisabeth Kübler-Ross. Following Kübler-Ross, Menning suggested that emotions need to change and go through specific changes; her approach arguably influenced the psychological study of infertility (Gameiro and Boivin 2017). In the newer adaptation of their handbook (1980), the Houghtons focused on matters beyond grief and provided more detailed steps on coping with infertility treatment and childlessness, as we will show below.

In Belgium, SARA was also aimed at supporting people in the emotional state of childlessness. While Dewickere and Franck organised separate meetings for couples who still hoped to have children through infertility treatment, their focus was on the acceptance of infertility and childlessness.22 The idea was that psychological recovery was dependent on active mourning. SARA’s work was rooted in the popular psychology literature of the time. In this light, SARA adopted Elisabeth Kübler-Ross’ stages of grief in the context of infertility: ‘It [involuntary childlessness] is a mourning process with all classic stages. You have to respect every stage. If it hurts, you have to say it. If you are sad, you have to say it’ (R.B 1991). SARA drew comparisons with other situations of grief and loss, including coping with the loss of a child, stillbirth and even disability—all topics for which similar self-help groups existed at the time. The work of the local self-help groups in these areas informed SARA’s work (Waes 1991).

Both SARA and the NAC focused on grief. The NAC drew attention to the specificities of infertility leading to childlessness with the idea of ‘unfocused’ grief. The Houghtons described is as ‘suffering from the sense of pointlessness and meaningless even despite having a partner and career, and loosing contact with people who have children even when peers’ (Houghton and Houghton 1977, 5). On the contrary, SARA represented the mourning process surrounding infertility as something that was comparable with other experiences of loss, such as the loss of a family member. This difference may be partially explained by how both groups positioned themselves in their respective national contexts. The Houghtons had presented the NAC as a national network of contacts in Britain. For Paul Dewickere and Frieda Franck, the role of SARA was to be embedded in a local network of support groups. These contextual differences also bring into question why, in the end, such similar approaches to emotions were raised around infertility; we address this below.

### Processing emotions: peer-to-peer counselling as non-directive counselling

Both the NAC and SARA represented grief as an active system in which emotions had to be ‘processed’. According to SARA, couples had to ‘work through’ their emotions by talking about them. Their publications stressed the need to ‘express your feelings’, to ‘talk about everything’ and ‘to not bottle up your feelings’ (Waes 1991). The presence of others was mainly to facilitate conversation between the spouses. SARA saw the priority of constant dialogue between the married couple as the central element of counselling. As they explained themselves in a recent interview:

‘By talking to third parties, you say things that you should perhaps have said to each other, but which are somewhat uncomfortable and slightly easier to mention to third parties. […] So once that difficulty in discussing it with each other is overcome, they can move on, and they don't need us anymore.’ 23

Within SARA, married couples were advised to utter all doubts and feelings, even if this meant hurting one another. It was not deemed wise to maintain silence to spare your partner’s feelings: ‘If you don’t speak, you [couple] drift apart’ (Het Nieuwsblad 1991, 14-15). The belief that talking is therapeutic and that silence within a couple indicates oppression underpinned this advice: Dewickere and Franck believed that ‘silent sorrow’ was by definition ‘unprocessed sorrow’.24

For NAC, it was also important to identify one’s emotions, such as feelings of loss, anger, jealousy and envy, by acknowledging them. However, unlike SARA, the primary focus of NAC was not on openness within the couple but on the interaction of the childless with the outside world (Houghton and Houghton 1977). First were interactions with loved ones. The Houghtons suggested that inquiry into one’s childlessness by family, friends or relatives caused intense feelings, which they saw as a problem that was only slowly being recognised. Second, they saw the need of the childless to share their experiences with other people in the same position. For example, NAC counsellors were seen as relevant because they themselves had been through the process of infertility (treatments), whether it had led to childbirth or childlessness. The role of infertility counsellors in this process, the Houghtons described as:

‘Counsellors need to be sympathetic and listen, they may suggest ideas for activities, but the most important and influential suggestions are likely to come from the childless person themselves’ (Houghton and Houghton 1984, 120–21).

This approach of letting counselees choose the direction of counselling is known as non-directive counselling. In marriage guidance, non-directive counselling has had a longer history (Wallis and Booker 1958). However, in the context of infertility, this idea was new, including for the Houghtons themselves, who introduced it in their 1984 handbook (Houghton and Houghton 1984). It is possible that the Houghtons adopted this view through the counselling training that NAC Contacts received; however, we were not able to trace any written accounts of these sessions. What is clear in their 1984 handbook, ‘*Coping with Childlessness’*, is that the Houghtons also used a step-by-step infertility journey as the guiding structure of the handbook. This emotions-focused and non-directive counselling approach to accepting one’s infertility through childlessness was similar to that adopted by SARA, as we show below.

### Destigmatising infertility and accepting childlessness

For both the NAC and SARA, the idea of the lived experiences of infertility as defining childlessness and childless people’s emotional state guided their approaches to counselling. In fact, counselling was necessary for the couple throughout their infertility treatments journey. The Houghtons described this process in their 1984 handbook as the ‘infertility treadmill’. The infertility treadmill was the process through which one—normally a couple—would have to go in their pursuit of parenthood (Houghton and Houghton 1984, 48). This included the medical side, where a husband and wife would be tested and told that they could not have children. The role of doctors and the way they spoke to the patient was also an important emotional aspect. The social side started with the postponement and delay of parenthood, which for some people may lead to infertility, and subsequent access to treatment and adoption, as well as the attitudes of friends and society to the childless. In this light, the process of facing these two sides was often traumatic, emotional, distressing and even depressing, according to the Houghtons.

The Houghtons’ own experience of infertility resembled this process. For them, the way out of the infertility treadmill was to find coping strategies. This was especially important because, as the Houghtons acknowledged, for the majority of the childless, medical treatments did not work, while donor insemination and adoption were not necessarily considered to be adequate or accessible alternatives to infertility (Houghton and Houghton 1984). Coping with infertility was, therefore, the only solution for these couples, and the Houghtons set out this vision as central to the NAC’s approach to infertility counselling. The important aspect of coming off the treadmill was, according to the Houghtons, ‘coming to terms with infertility and seeking another yardstick by which to measure the success of one’s life’ (Houghton and Houghton 1984, 71). This meant that the grief had to go away in order for a childless person to face a life anew. To do so, the childless person had to make a conscious decision to terminate medical inquiries, including around donor insemination and adoption. The Houghtons recognised that this was the hardest part of the coming-to-terms process—‘stopping treatment at a particular point at their own free will’—which, they suggested, was hard not only for couples but also single women who were trying donor insemination (Houghton and Houghton 1984, 12). It is here that the role of non-directive counselling in helping couples to find this moment was vital.

For SARA, too, the importance of the grief and loss perspective was related to the low treatment success rates of the time, with an—often implicit—assumption that most people trying treatment would eventually have to cope with remaining childless (Stuckens 1992). As such, the focus of SARA was on accepting infertility and the notion that acceptance could only be reached through active mourning. However, before one could start the mourning process, one had to let go of any hope of having children and as such make a conscious decision to stop any treatments. Even though they did not use the metaphor of the treadmill, the discourse of SARA was quite similar to that of the NAC. In a newspaper article with the telling subtitle ‘Self-help group SARA helps you out of an inevitable spiral of hope, disillusionment and sadness’, Dewickere and Franck explained:

‘The decision [to quit treatment] does not only bring pain, but also joy. For only from that moment could we allow ourselves to grieve. As long as you go from one waiting room to another – for adoption or for IVF – you live under great tension. One day you are full of hope and expectation, the next day it is all shattered. But you cling to the next attempt, you give yourself no rest. Years pass without you being able to process your grief’ (Stuckens 1992).

Much like the NAC, SARA urged couples seeking infertility treatments to look at medical solutions critically and to understand that they could keep couples in a constant state of (false) hope. In general, Dewickere and Franck were rather sceptical about infertility treatments. In an interview in 1993, they described IVF as an ‘experiment’, the low success rates of which did not justify the heavy psychological burden. In their own words: ‘We were given a 20 per cent chance of success […] The chances of conception were too small and the mental suffering too great.’ (Waes 1993, 27). Again, this followed from their personal experiences. As Frieda Franck also explained in a recent interview, they had tried IVF once, but decided to quit because she ‘found the insecurity harder to bear than the security of remaining childless’.25 In her view, it was ‘mentally unhealthy to keep waiting and trying’, because it kept one from ‘accepting it and making the most of your life’.26

In the views of SARA and the NAC, it was only after ‘having come to terms’ with infertility that the childless could find new meanings in life. SARA stressed that people could be ‘life-giving’ in ways other than raising children: ‘childless people may actively search for their own ways to be “fertile”.’ For example, they suggested that they could care for those in need, such as the disabled or the elderly, or that they could ‘put [their] energy in a third or fourth world movement, in a socio-cultural association, in a sports association, and so on’ (Dewickere and Franck 1994, 78). In the view of the Houghtons, abandoning hope was a necessary step for achieving a reconciliation and eventually coming ‘full circle’, which they called the ability to accept the change. For the NAC, the way to do this was to take on new activities, such as getting a pet, as well as stopping some things that may trigger childlessness-related emotions. In their handbook, the Houghtons also suggested that the Western notion of the nuclear family and the role that children play in it also had to be reconceptualised as part of this process of counselling. To what extent this was done in practice is a question for a more detailed further study.

## Discussion and conclusions

We have argued in this article that the work of the self-help support groups in Britain and Belgium set infertility counselling in motion. Both groups adopted a peer-to-peer counselling format provided through local support groups. Due to their different political contexts, the ways in which these groups were set up in Britain and Belgium varied. The NAC in Britain had local connections to the Birmingham Settlement and quickly became a national network. Similar to other sexual and reproductive health counselling initiatives in Britain, NAC was also established as a secular organisation at the grassroots level. The NAC was arguably the first and only initiative in Britain that identified the need for and started to provide infertility counselling before the British Infertility Counselling Association was set up in 1988 (Jennings 1995). In Belgium, the first groups that offered self-help around infertility were established in 1983 from a general and more centralised initiative by Trefpunt Zelfhulp. Although they were set up in several places in the country, these self-help groups were short-lived. SARA, in turn, was a more long-lasting self-help initiative that developed a distinct approach to infertility counselling. Although open to anyone, SARA was influenced by the Catholic pillar in Belgian society. In Belgium, there were more professional efforts to establish infertility counselling, for example by the CNP in the beginning of the 1990s; while they were not linked to SARA or other support groups directly, they were indeed inspired by those organisations and used similar methods as other support groups.

Set up nearly a decade apart in distinct political contexts, the NAC in Britain (1976) and SARA in Belgium (1989) nevertheless developed similar approaches to infertility counselling. They grounded their counselling approaches in popular psychology literature of the psychologist Elisabeth Kübler-Ross in one way or another. Because these peer encounters were grounded in specific psychological literature of the time, it permitted the provision of systematic counselling, which ad hoc self-help groups would be unable to provide. Both groups saw grieving and mourning as important stages of controlling and overcoming the negative and frustrating emotions that the involuntarily childless encounter when going through infertility treatments or indeed when making the decision to stop unsuccessful treatments and accept childlessness. The role of systematic peer support—what is today known as peer-to-peer counselling—was to help couples to go through those different emotional stages of their infertility treatments in their counselling sessions: processing the emotions of grief and mourning, destigmatising infertility, and accepting childlessness. Crucially, both NAC and SARA departed from the importance of childless people’s belonging in a ‘child-oriented society’, which they believed dominated both British and Belgian societies at the time. Therefore, this emotional guidance through peer-to-peer counselling aimed at destigmatising infertility at large.

The founders of NAC and SARA informed these very similar approaches to emotional guidance through their personal struggles with infertility as married couples. Diane and Peter Houghton in the NAC and Paul Dewickere and Frieda Franck in SARA—all themselves childless due to infertility—founded these respective organisations with a view to helping other people to deal with infertility treatments and involuntary childlessness. We highlighted that NAC’s approach of coping with childlessness seemed more individualistic; it was focused on personal solutions of coming to terms with the situation. Meanwhile, SARA’s approach, which was strongly focused on married couples and suggested the childless look for a new purpose to serve society, can be seen as more society-oriented and rooted in Catholic teaching. Because we focused only on the founding figures and less on other members of these initiatives, it is unclear what role race, sexuality and class, played in shaping emotions around infertility within these support groups and beyond them. To better understand the emotional norms that these groups created around infertility, a more detailed investigation beyond the founding figures is necessary. Furthermore, studies might aim to trace the ways in which secular and religious values shaped emotional regimes of self-help groups in reproductive health.

Another crucial point of NAC’s and SARA’s approaches was to disassociate the feelings of hope, which at that time was becoming strongly associated with medical fertility treatments, from the emotional process of accepting childlessness. This distinction has largely gone unnoticed by historians so far. Indeed, this distinction became overshadowed by the idea of infertility treatments giving hope (Greil 1991) and eventually becoming ‘hope technologies’ (Franklin 1997; Inhorn *et al*. 2022). Nevertheless, these first infertility support groups warned against infertility treatments becoming ‘hope technologies’. As such, those alternative narratives of infertility, and indeed emotional norms around them, need to be further investigated. Finally, all the developments we describe in this article took place before infertility counselling was recognised as a profession in the late 1980s and early 1990s. The practitioners and independent organisations working in this field of medicine today should be attuned to cultural contexts of emotions that existed alongside the infertility treatments in the recent past.

## Data Availability

Data sharing is not applicable as no data sets were generated and/or analysed for this study. All the archival materials are available from the libraries and archives as specified in the paper. The four oral history interviews conducted by the authors are not available to consult (ethical approval was secured for the purposes of the aforementioned projects).

## Notes

1. While infertility and involuntary childlessness today refer to distinct social experiences, the support groups in this study perceived the former to result in the latter: infertility leading to involuntary childlessness.

2. Patients or the public *were not* involved in the design, or conduct, or reporting, or dissemination plans of our research.

3. Companionate marriage was a representation of ‘romantic, intimate and egalitarian’ couple relationships (Fisher 2013, 329).

4. These were the Centres for Birth Control and Sexual Education (CGSO, Centra voor Geboorteregeling en Seksuele Opvoeding) and the National Centre of Pastoral Care of the Family (CNPF, Centre national de pastorale familiale), renamed the Centre of Education regarding the Family and Love after the publication of Humanae Vitae in 1968 (CEFA, Centre d’éducation à la famille et à l’amour).

5. For example, the archives of CGSO, inventory numbers 3.5 and 8.1, kept in AMSAB (Institute of Social History) in Ghent. Infertility was also occasionally mentioned in the yearbooks of the CGSO (eg, Temmerman 1985).

6. Dossier: l’insémination artificielle, CEDIF (Centre de documentation et d’information de la Fédération Belge pour le Planning Familial et l’Education Sexuelle), Janvier 1982, kept in AMSAB (Institute of Social History) in Ghent.

7. Flemish Radio and Television Archives (VRT) archive, AIM01279498.

8. Peffer (1993, 160–61) and Pfeffer and Woollett (1983, 40–56) suggest that some doctors had a selection procedure for which patients would have access to infertility treatments, and other studies also show that it was especially the cases for donors insemination (Davis 2015; Snowden and Mitchell 1981).

9. The situation with infertility counselling is similar to that around abortion counselling in Britain. As scholars suggest (Hoggart 2015), abortion counselling originally only implied advice on making a decision, and did not include emotional guidance.

10. Report of the first discussion afternoon for insemination centres, Leiden, 18 May 1979, Archives of Omer Steeno, University Archives of Leuven.

11. Oral history interviews with Marilyn Crawshaw on 8 May 2019 and Jim Monach on 15 May 2019.

12. Oral history interviews with Jim Monach on 15 May 2019.

13. Originally, NAC was set up as an association for both the childless and the child-free. However, the latter split from NAC in 1978 (Birmingham Settlement 1979).

14. Lists of all self-help groups can be found in the publication *Sociale kaart van de zelfhulporganisaties en-groepen in Vlaanderen*, which appeared regularly in the 1980s and 1990s.

15. Poster Alice et les trois E, undated, Affiches, nr. AA 1503, AVG Carhif, Brussels.

16. According to the CNP, ‘Pauline et Adrien’ counted 700 Belgian members in 1993. See: Dossiers relatifs aux groupes de paroles organisés pour les couples infertiles, 1989–1997, Archives CNP, nr. 33, AVG Carhif, Brussels.

17. Dossiers relatifs aux groupes de paroles organisés pour les couples infertiles, 1989–1997, Archives CNP, nr. 33, AVG Carhif, Brussels.

18. This section is based on oral history interviews with the founders of SARA, Dewickere and Franck, on their personal archives, and on their book De stille tuin (Dewickere and Franck 1994).

19. Oral history interview with Paul Dewickere and Frieda Franck, 19 February 2019.

20. For example, this emphasis on the well-being of couples, rather than individuals, was also an important characteristic of the counselling offered by the ‘communication centre for spouses’ at the Catholic University of Leuven (Verhulst and Vansteenwegen 2008).

21. The NAC recognised Resolve as their sister organisation (kNACk 1978), but little research has been undertaken on the extent of those connections.

22. Oral history interview with Paul Dewickere and Frieda Franck, 19 February 2019.

23. Oral history interview with Paul Dewickere and Frieda Franck, 19 February 2019.

24. Oral history interview with Paul Dewickere and Frieda Franck, 19 February 2019.

25. Oral history interview with Paul Dewickere and Frieda Franck, 19 February 2019.

26. Oral history interview with Paul Dewickere and Frieda Franck, 19 February 2019.
